# The love and hate relationship between cellular senescence and stemness

**DOI:** 10.18632/aging.206387

**Published:** 2026-05-30

**Authors:** Angelos Papaspyropoulos, Giorgos Theocharous, Orestis A Ntintas, Konstantinos Evangelou, Vassilis G. Gorgoulis

**Affiliations:** 1 Molecular Carcinogenesis Group, Department of Histology and Embryology, Medical School, National and Kapodistrian University of Athens, 11527 Athens, Greece; 2 Biomedical Research Foundation, Academy of Athens, 11527 Athens, Greece; 3 Intelligencia Inc, New York, NY 10014, USA; 4 Division of Cancer Research, Ninewells Hospital and Medical School, University of Dundee, DD19SY Dundee, UK; 5 Faculty Institute for Cancer Sciences, Manchester Academic Health Sciences Centre, University of Manchester, Manchester M20 4GJ, UK

**Keywords:** senescence, stemness

## Abstract

Cellular senescence and stemness represent two biological entities with several opposing properties. However, both senescence and stemness serve to maintain tissue homeostasis, via different mechanisms. In adults, while non-dividing senescent cells represent a major barrier to potentially harmful cell insults, propagating stem cells are responsible for restoring the structure and functionality of damaged tissue. In this review, we highlight distinct cellular settings where an antagonistic relationship between the two states is naturally established. In contrast, major synergy between senescence and stemness is observed primarily in cancer, where inherent senescent cell features may actively promote the emergence of cancer stem cells. As the complex interplay between senescence and stemness may heavily vary between different cell types and physiological contexts, elucidating the nature of the interaction and the potential effects per case, is of considerable clinical importance.

## INTRODUCTION

Cellular senescence is defined as a stress response mechanism characterized by a prolonged and stable cell cycle arrest, principally constituting a homeostatic mechanism against a variety of cellular insults [1, 2]. This conventional view of senescence comes in contrast with a basic component of stem cell biology, that of self-renewing propagation and subsequent differentiation compensating for tissue damage and cell loss [3]. Despite, however, the evident opposite functions of senescence and stemness with regards to cellular proliferation, accumulating data keep pointing to a gray zone where senescence, may in fact, promote stemness depending on the cell type and biological context. Given the context-dependent relationship between senescence and stemness, here we provide an overview of the so far documented cellular and clinical settings where antagonism or synergy between the two entities has been elucidated, both at the molecular and functional level. To that end, we outline the cell/tissue types where distinct molecular regulations have been identified to block or enable stem or senescent cell traits and functions, through key molecular player or pathway interactions. We additionally provide the biological frame where each regulation may apply; either in homeostatic conditions or disease settings, for example cancer.

## The characteristics of cellular senescence

Based on its triggers and inherent features, senescence is completely distinct from terminal differentiation and quiescence [2]. So far numerous stressors have been identified to induce senescence, including genotoxic agents, hypoxia, nutrient depletion, mitochondrial damage as well as oncogene activation [1]. Senescent cells acquire a unique secretome termed Senescence-Associated Secretory Phenotype (SASP), accompanied by characteristic morphological and functional changes due to alterations in cellular shape and genetic/epigenetic regulation, as well as extensive metabolic rewiring [1, 2, 4]. It is already well established that transient induction of senescence is beneficial for retaining organismal homeostasis, as it contributes to immune system-mediated clearance of dysfunctional cells, a process of paramount importance for embryogenesis and wound healing [1, 2, 5, 6]. On the other hand, the prolonged presence of senescent cells is linked with chronic inflammation, setting the grounds for age-related disease such as cardiovascular and neurodegenerative disorders and cancer [1, 7–10].

Senescence can be triggered either through the natural exhaustion of the replicative potential of the cell, reflected in telomere attrition (replicative senescence) or prematurely, in response to telomere-independent stressors such as oxidative stress, oncogene activation and drug treatment (collectively referred to as stress-induced senescence) [1]. Senescence is a highly heterogeneous process, displaying differential regulation depending on the stimulus [11–13]. However, despite the versatile and dynamic establishment of senescence across various settings, senescent cells display four global hallmarks: (i) cessation of proliferation, (ii) acquisition of critical metabolic adaptations, (iii) macromolecular damage and (iv) SASP manifestation (Figure 1A) [1, 4]. Importantly, it has been observed that early senescence, which is characterized by an inherently transient cell cycle arrest, subsequently shifts towards a stable proliferation halt, as early senescent cells eventually undergo “deep” senescence upon proper SASP establishment [13].

**Figure 1:**
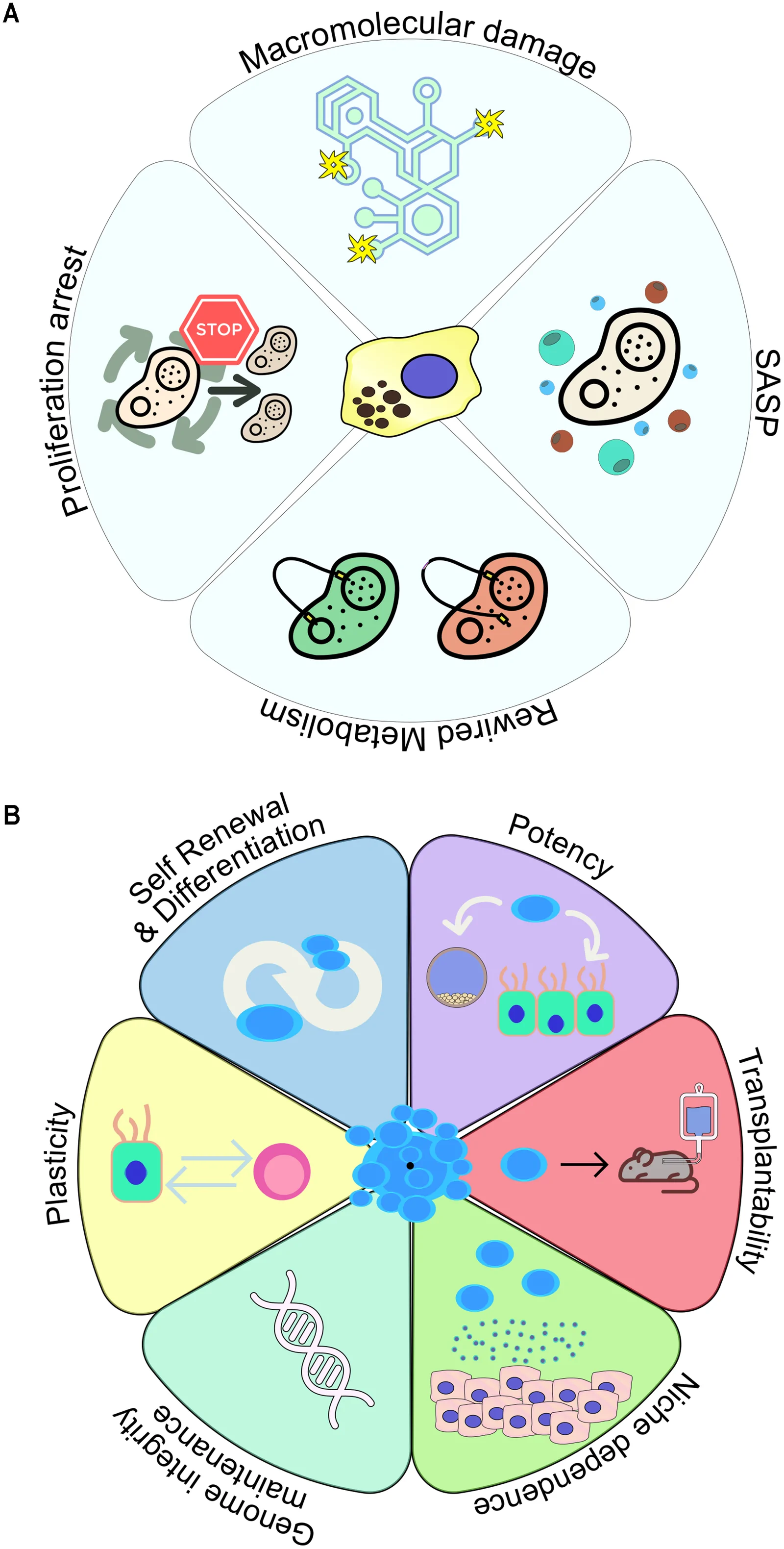
Key hallmark characteristics of (**A**) senescent and (**B**) stem cells.

Molecularly, senescence-mediated cell cycle arrest requires activation of the p53/p21^WAF1/Cip1^ and p16^INK4A^/RB axes, where the transient induction of p21^WAF1/Cip1^ expression is regarded as critical for the initial acquisition of the senescent phenotype [1, 14–17]. Maintenance of the senescent phenotype, however, appears to rely on stable p16^INK4A^ expression within the senescent cell [1, 14]. Additionally, DNA damage in senescent cells presents in the form of double-strand breaks, which in turn engages the DNA damage response (DDR) pathway, as indicated by the formation of persistent DDR foci [18–21].

More recent evidence suggests that under defined conditions, a possible reversion of the senescence state may occur, followed by complete resumption of proliferation, a process termed “escape from senescence” [22–27]. Specifically, a number of studies have demonstrated that upon oncogene-mediated induction of senescence (oncogene-induced senescence), regardless of the epithelial or mesenchymal origin of the cells, those may “escape” the senescent state and resume proliferation, accompanied by aggressive oncogenic traits [1, 22, 25, 28–30].

One of the most prominent phenotypes of senescent cells is the development of a flattened, enlarged morphology, partly indicating the extensive adaptations occurring upon senescence induction [1, 2, 4]. Senescent cells do not only reprogram their metabolism, but additionally exert paracrine activity by releasing factors that impact the fate and function of neighboring cells. This capacity is widely attributed to SASP, consisting of pro-inflammatory cytokines, inflammation inducers and growth factors [31–34]. As with transient and chronic senescence, early SASP holds a beneficial role in tissue and organismal homeostasis, as it contributes to the recruitment of immune cells which act to remove senescent cells throughout the processes of wound healing and tissue growth. On the contrary, a persistent SASP gradually acquires a pro-inflammatory role, thus facilitating the onset of senescence-related disease [11, 13].

Besides telomere attrition and accompanying DNA damage, common types of macromolecular damage within senescent cells pertain to protein and lipid oxidation [35, 36]. Macromolecular damage is responsible for the increased release of reactive oxygen species (ROS), with detrimental effects on protein aggregation and misfolding [37]. This level of extensive macromolecular damage occurs in parallel with deregulation of metabolic pathways controlling pivotal cellular processes including glycolysis, autophagy and mitochondrial function, altogether driving various senescence-related metabolic disorders [38]. In the context of macromolecular damage and deregulated metabolism, lipofuscin accumulation (the “dark matter” of senescent cells) constitutes the most stable component of senescent cells [1, 39]. Lipofuscin composition is not uniform, as it consists of a heterogeneous aggregate of damaged macromolecules - including oxidized proteins, lipids and metals - whose relative abundance may differ depending on the cellular context [39]. Fluorescent lipofuscin aggregates in senescent cells have been exploited for the generation and utilization of novel senescence-detection tools [40–42]. These reagents have been already integrated in detection algorithms and applied in various senescence detection and elimination settings [43–48]. It needs to be stressed that current consensus on senescence detection requires a multi-marker approach and context-aware assessment [44].

## Overview of stemness

All tissues undergo a natural rate of cellular turnover due to physiological wear and tear. In most cases lost cells are replenished through the function of stem cells, whose properties may vary between different types of tissues; however the landmark feature of stem cells is their ability to coordinate cell division and differentiation in order to eventually repopulate the tissue (Figure 1B) [3]. Stem cells are present both in embryos (embryonic stem cells) and adults (adult stem cells). Their differentiation potential peaks in the beginning of embryogenesis, where totipotent stem cells give rise to all types of progenitor cells in the organism [49]. Pluripotent stem cells (PSCs) are derived from the inner cell mass of the embryo and differ from totipotent stem cells only in their inability to form extraembryonic structures (e.g. placenta), whereas multipotent stem cells, residing in adult tissue, have an even more limited differentiation capacity [49]. An example of multipotency is the hematopoietic stem cell, which can differentiate into various blood cell types, including unipotent stem cells which display the narrowest differentiation potential as they can only form one terminally differentiated cell type (e.g. erythrocytes) [49].

Induced pluripotent stem cells (iPSCs) represent a distinct category of stem cells, as they are generated via artificial reprogramming of somatic cells towards an embryonic-like state, thereby closely resembling PSCs [50]. Somatic cell reprogramming into iPSCs traditionally requires the ectopic expression of a transcription factor mix (OCT4, SOX2, KLF4, MYC – collectively referred to as OSKM) [50–52]. The process of reprogramming is accompanied by extensive remodeling of the epigenome as well as profound alterations in critical biological circuits involved in metabolism, proteostasis and cellular signaling [50, 53–55]. Despite the limitations of iPSCs which are inherently connected to the reprogramming process *per se*, such as accumulation of tumorigenic mutations [56, 57] or maintenance of “epigenetic memory” linked to their previous somatic cell identity [57–60], iPSCs have been already applied in human development and disease modeling, and their versatility led to their utilization as drug screening and cell therapy platforms [50]. Some of the limitations of the reprogramming technology have been alleviated by the development of patient-derived organoids (PDOs) directly from adult stem cell pools. Upon exogenous differentiation cues provided as cell medium components, complex 3D structures are generated *in vitro*, where tissue architecture and various cell type interactions are more accurately represented [57, 61–63].

Due to their functional purpose, stem cells exhibit a unique proliferation pattern. For decades, researchers have referred to the hematopoietic system to describe the principles of stem cell biology, characterized by a hard-wired hierarchy where an initial stem cell asymmetrically divides to give rise to its progeny [3]. Asymmetric cell divisions represent the established model to ensure indefinite survival of a stem cell population [64]. Upon dividing asymmetrically, intracellular fate determinants or external differentiation cues guide each stem cell to produce two different types of offspring; an equally potent stem cell and a differentiated progenitor [64]. However, asymmetric cell divisions must be replaced by exclusively symmetric ones (where each division produces two identical stem cells), in cases where stem cell exhaustion is observed [3, 65, 66]. Quiescence (a cellular state characterized by a prolonged and generally reversible proliferation exit) has been traditionally perceived as an inherent trait of stem cells, helping avoid stem cell exhaustion and minimize the risk of accumulating DNA mutations through an increased number of replication cycles in a lifetime [67].

For several years, the gold standard in stem cell identification was their capacity to propagate and differentiate following their transplantation into animal models. Based on this property, single stem cells are capable of yielding functional mammary tissue a few weeks after their engraftment in the fat pad [68, 69]. Similarly, single satellite cells are able to produce whole muscle fibers containing thousands of cells [70]. It has been established now that stem cells are able to adapt well to continuously changing environmental signals to produce progeny with high efficiency because of their extensive plasticity [3]. For example, in the gastrointestinal tract, in response to damage, stem cells can change their cellular fate; crypt base columnar (CBC) cells are depleted upon chemotherapy, irradiation or genetically, however they can be sufficiently regenerated through the emergence of new CBCs from a number of non-CBC pools [71, 72].

Identifying the niche factors determining the balance between stem cell self-renewal and differentiation remains a challenge, as the source and type of those signals may significantly vary depending on the tissue type. Along these lines, it is important to delineate the mechanisms through which stem cells maintain their genomic integrity, which is crucial in preventing tumorigenic growth or irreversible loss of tissue [3]. Interestingly, some stem cell populations have been found to orchestrate the DNA repair machinery differently compared to their committed progeny [73]. One such example is mammary gland stem cells, which appear to display augmented levels of Non-Homologous End Joining (NHEJ) activity than their committed daughter cells [74]. In adult stem cells (e.g. liver and gut), telomere attrition is prevented by increased expression of telomerase [75, 76], while the accumulation of mutations can be prevented through distinct metabolic adaptations [3]. For instance, in hematopoietic stem cells a considerably low glycolytic activity was observed, leading to restriction of ROS generation, which would otherwise contribute to DNA damage [77].

A distinct and clinically important entity in stem cell biology are cancer stem cells (CSCs), a subset of cells within tumors acquiring stem cell features including continuous self-renewal, ultimately leading to oncogenic growth and metastasis, while contributing to tumor heterogeneity [78]. The process driving CSC formation is known as dedifferentiation, through which differentiated cells regain stem cell properties, such as pluripotency and self-renewal [79–81]. In general, CSCs and normal stem cells are functionally alike, even displaying similar transcriptional profiles [80]. Apart from self-renewal and pluripotency, which have been regarded essential traits of CSCs since their discovery in 1997 [82], activation of the Wingless- type (Wnt) signaling pathway has been also considered key in driving CSC formation and growth [79]. Several other players may also contribute to the regulation of CSC formation, such as loss of Sterile Alpha Motif Domain (Smad4), which together with Wnt pathway activation has been found to promote the return of the intestinal epithelium to a stem-like state, thus initiating cancer growth [83]. Similarly, aberrant Wnt pathway activation is also linked to dedifferentiation of breast cancer bone metastatic cells into CSCs [84].

## Antagonistic interplay of senescence and stemness

Several cellular settings have been identified where senescence induction seems to exclude stem cell activity, thereby suggesting a functional competition between the two processes (Figure 2). One such example are mesenchymal stem cells (MSCs), which on account of their multipotent nature have been considered promising vehicles in tissue regeneration and other clinical endeavors, such as cartilage healing and immune response modulation [85–88]. Many of the potentially beneficial MSC properties are attributed to their ability to grow as high-density monolayers known as MSC sheets [87, 88], which however can rapidly acquire senescence features under sustained high-density conditions [89, 90]. The mechanistic Target Of Rapamycin (mTOR) is a master regulator of cellular processes such as growth and survival, protein synthesis and lipid metabolism [91]. It was shown that rapamycin, the well-known inhibitor of mTOR, increased autophagy in placental-derived MSCs (PMSCs) and blocked senescence induction, which was accompanied by a decreased cellular size [92]. Thus, rapamycin was proposed as a potential strategy in preventing senescence and expanding the utility of MSC sheets [92]. IL-8 is a chemokine playing an important role in the attraction of pro-healing immune cells (e.g. macrophages and mast cells) to sites of wound healing in order to facilitate the process [93, 94], and rapamycin was found to increase IL-8 levels in damaged MSC-derived osteoprogenitor cells, indicating again its implication in boosting MSC fitness [92]. It should be noted, however, that IL-8 may be also implicated in chronic inflammation, which renders its activity context-dependent [95, 96]. Of note, with regards to wound healing, it was also shown that senescence of alveolar epithelial progenitor cells impairs the lung regenerative capacity and drives fibrosis [97, 98].

**Figure 2:**
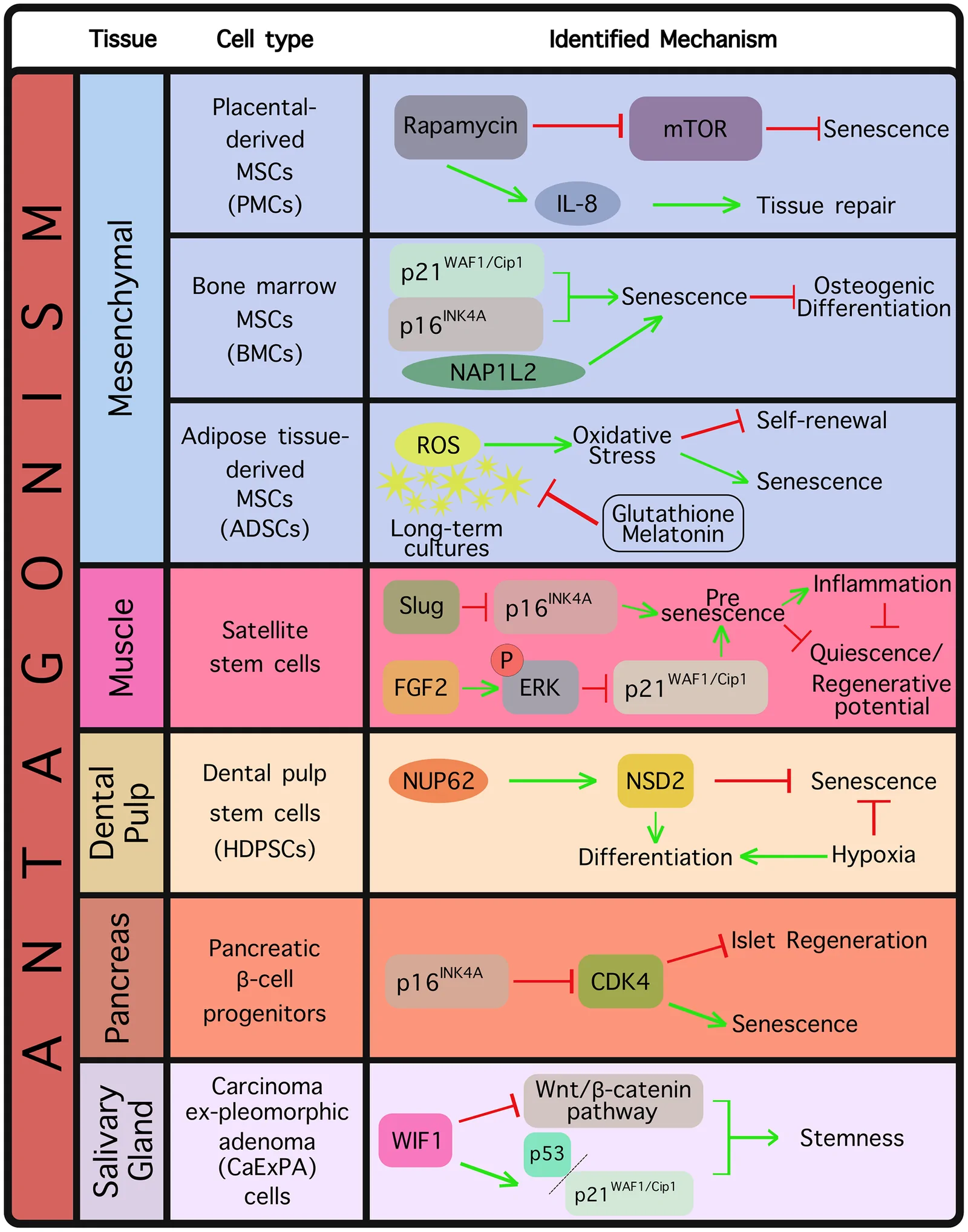
**Tissue and cell types where an antagonistic relationship between senescence and stemness has been already documented.** The identified mechanism(s) are displayed in each case.

Another case of functional competition between senescence and stemness relates to bone marrow mesenchymal stem cells (BMSCs) which constitute a lifelong reservoir for somatic cell generation, and are shown to be significantly useful in bone regenerative medicine [99, 100]. It has, however, been observed that BMSC osteogenic differentiation is inhibited by senescence, thereby limiting the BMSC regenerative potential [101]. In recent work, Hu et al. (2022) derived BMSCs from elderly donors and confirmed a robust establishment of senescence compared to young counterparts, based on a series of observations, including upregulation of p21^WAF1/Cip1^ and p16^INK4A^ levels, G1 phase cell cycle arrest and increased telomere DNA damage, accompanied by inhibited differentiation potential [102]. Interestingly, they also identified the *NAP1L2* gene, a critical factor in mouse neuronal differentiation [103, 104] as an important regulator of senescence, as its depletion prevented BMSCs from entering senescence and resulted in a diminished SASP profile [102]. Thus, NAP1L2 emerged as a novel switch in driving MSC senescence and suppressing osteogenic differentiation [102].

Adipose tissue-derived mesenchymal stem cells (ADSCs) constitute valuable candidates in treating a number of regenerative disorders, as apart from being multipotent, they have been shown to be involved in immunomodulation and display strong paracrine activity [105, 106]. ADSCs lose their primitive stemness easily, accompanied by reduced proliferation activity and emergence of senescence upon long-term expansion of *in vitro* cultures [107], which has been largely attributed to a disturbed redox homeostasis [108, 109]. Specifically, abnormally high production of ROS contributes to oxidative stress, negatively affecting stem cell self-renewing and differentiation properties and establishing stem cell senescence in long-term cultures [108, 110]. In a study by Liao et al. (2019), ADSCs received low doses of the reduced glutathione and melatonin antioxidants, which led to maintenance of ADSC functions by limiting senescence and fostering cellular migration, through inhibiting ROS production [111]. Most importantly, antioxidant-treated ADSCs continued to display multidirectional differentiation properties [111].

A considerable body of evidence where documented antagonism between senescence and stemness is pivotal for physiological homeostasis refers to muscle tissue. Muscle tissue regeneration relies heavily on satellite cells, a quiescent adult stem cell population whose regenerative capacity declines upon aging [112]. Sousa-Victor et al. (2014) reported that satellite cells from geriatric populations fail to retain their quiescent state under normal conditions, which extensively impacts their self-renewal and regenerative properties [113]. The authors found that resting satellite cells in geriatric mice switch to a pre-senescence state thereby losing quiescence, a process driven by *p16^INK4A^* derepression [113]. They additionally showed that upon injury, those cells fully enter senescence, which is rescued upon p16^INK4A^ silencing as evidenced by restoration of quiescence and their regenerative potential [113]. Those data demonstrate that maintenance of quiescence in adults, in fact, relies on repression of senescence [113]. Along these lines, more recent evidence highlighted the transcription factor Slug (a known Epithelial-To-Mesenchymal transition (EMT) marker) as a *p16^INK4A^* repressor in satellite cells [114]. Specifically, it was found that Slug loss triggers satellite cells to acquire pre-senescence features driven by upregulation of p16^INK4A^, which was followed by significant regenerative defects upon muscle damage [114]. In accordance with previous findings [113], it was reported that Slug reduction and p16^INK4A^ upregulation in satellite cells occur naturally in the process of aging, however restoration of Slug levels is sufficient to rejuvenate aged satellite cells, indicating that Slug may be an important player in age-related muscle degeneration [114].

In order to explain the proliferative decline of satellite cells over time, Li et al. (2015) suggested the presence of increased epigenetic silencing on the *p21^WAF1/Cip1^* and *p16^INK4A^* genetic loci in young compared to old muscle [115]. The authors additionally found that ectopic Fibroblast Growth Factor 2 (FGF-2) expression induced extracellular signal-regulated kinase (ERK) phosphorylation resulting in *p21^WAF1/Cip1^* epigenetic silencing, a mechanism potentially capable of affecting the fate of old muscle cells. Moreover, the authors showed that other cyclin-dependent kinase inhibitors (CDKIs), such as p15^INK4B^ and p27^KIP1^ displayed incremental expression in satellite cells over time, thereby contributing to the aging satellite cell regenerative defect [115].

In a recent study conducted by Moiseeva et al. (2022), various senescent cell types were isolated from young and old mouse damaged muscle, via SPiDER-βGal, a flow cytometry-based approach relying on SA-β-Gal expression [116]. The isolated cells were subsequently subject to a set of downstream analyses, including transcriptome and chromatin analyses, overall yielding a senescent cell atlas in which inflammation emerged as a key factor potentially blunting muscle regeneration [116]. In particular, senescent cells were shown to comprise an inflamed niche mirroring aging-associated inflammation [117], appearing responsible for arresting stem cell proliferation and inhibiting regeneration. Importantly, by restricting the senescent cell burden or the inflammatory secretome, regeneration was facilitated in both young and old mice, whereas senescent cell transplantation conferred the opposite effects [116].

A recent proteomic analysis in human dental pulp stem cells (HDPSCs), retrieved from individuals belonging in various age groups, identified NUP62, a component of the nuclear pore complexes (NPC) as a highly downregulated factor in aged HDPSCs [118]. NUP62 is located in the central NPC avenue, holding a critical role in selective transport regulation between the nucleus and cytoplasm [119]. NUP62 was subsequently shown to restrict senescence-associated phenotypes, while enhancing the differentiation capacity of HDPSCs *in vitro* and *in vivo*, effects that were completely reversed upon NUP62 silencing [118]. Interestingly, the authors found that NUP62 represses senescence in HDPSCs by eliciting the nuclear transport of the E2F1 transcription factor, leading to the transcriptional upregulation of the Histone-lysine N-methyltransferase NSD2, which acts as an epigenetic reprogramming factor favoring stemness [118]. Another study attempted to investigate the interplay between stemness and senescence again in HDPSCs, this time in response to hypoxia [120]. Interestingly, the authors showed that although normoxic conditions over time led to loss of stemness and establishment of senescence, those effects could be rescued upon switching into hypoxia. Along these lines, it was shown that stemness markers such as STRO-1 and OCT4 were upregulated in hypoxic conditions, accompanied by a reduction in the number of senescent cells and downregulation of senescence-related genes [120].

Besides functioning as a senescence effector, p16^INK4A^ is a potent inhibitor of the known cell cycle regulator cyclin-dependent kinase 4 (CDK4) [121], which is indispensable for proliferation and differentiation of pancreatic β-cells in mammals [122, 123]. It was found that p16^INK4A^ limits islet regeneration with age, and that p16^INK4A^ expression is elevated in the islets compared to the exocrine pancreas [124]. Importantly, using mouse models, the authors noted decreased survival with age following depletion of β-cells, an expected outcome given that islet proliferation is instrumental to survival; however, *p16^INK4A^*-lacking mice exhibited pronounced islet proliferation and increased survival following β-cell ablation [124]. The authors linked p16^INK4A^ expression to induction of senescence in β-cells or their progenitors [124].

The *Ink4/Arf* genetic locus, which is a known driver of senescence, has been found to also act as a barrier for iPSC reprogramming in a cell autonomous manner [125]. Genetically blocking or reducing *Ink4/Arf* expression increases both the efficiency and speed of reprogramming. Additionally, Banito et al. (2009) have attributed slow reprogramming rates to barriers raised as a consequence of the forced expression of the OSKM reprogramming factors [126]. Senescence was identified as one such barrier, impairing successful reprogramming to pluripotent stem cells through upregulation of p53, p16^INK4A^, and p21^WAF1/Cip1^ [126].

As mentioned in the previous section, the Wnt/β-catenin signaling pathway is a pivotal signaling cascade actively controlling the balance between cellular proliferation and differentiation, a critical function for embryogenesis, maintenance of adult tissue homeostasis and tumorigenesis [127–129]. It is known that Wnt signaling holds an important role in cancer development, partly via its contribution to the emergence of cancer stem cells (CSCs) [130, 131]. In keeping with this, Wnt inhibitory factor 1 (WIF1) was identified as a Wnt pathway antagonist, binding to Wnt ligands and inhibiting their recognition by the respective receptors, resulting in β-catenin degradation [132]. It was reported that WIF1 decrease is a frequent event in salivary gland carcinoma ex-pleomorphic adenoma (CaExPA), and WIF1 upregulation in salivary gland cancer cells abrogated stemness-related phenotypes thereby promoting senescence, possibly via activation of p53 and p21^WAF1/Cip1^ [133].

## Functional synergy between senescence and stemness

Although competition between senescence and stemness has been observed in several tissue types under physiological conditions such as the muscle, bone marrow, adipose tissue and pancreatic islets, functional synergy is mostly encountered in the context of tumorigenesis (Figure 3). It appears that stemness-associated senescence could initially mask emerging stem cell features within a senescent cell, however those features may ultimately contribute to the “escape from senescence” phenomenon. As mentioned above, in cancer cells, acquisition of stemness may dramatically impact tumor aggressiveness, and ultimately, clinical outcome. Milanovic et al. (2017) implemented Eμ-*Myc* transgenic mice developing B-cell lymphomas to explore whether cancer stemness could be regulated by chemotherapy-induced senescence, and indeed found that senescent lymphomas displayed a robust stem cell signature, accompanied by expression of stemness markers and an active Wnt pathway [134]. Moreover, the authors showed that cells escaping senescence resumed proliferation exhibiting augmented Wnt-dependent growth compared to cell counterparts that had never entered senescence following chemotherapy [134]. In line with this, cells escaping senescence displayed a significantly increased tumor initiation potential *in vivo* [134].

**Figure 3:**
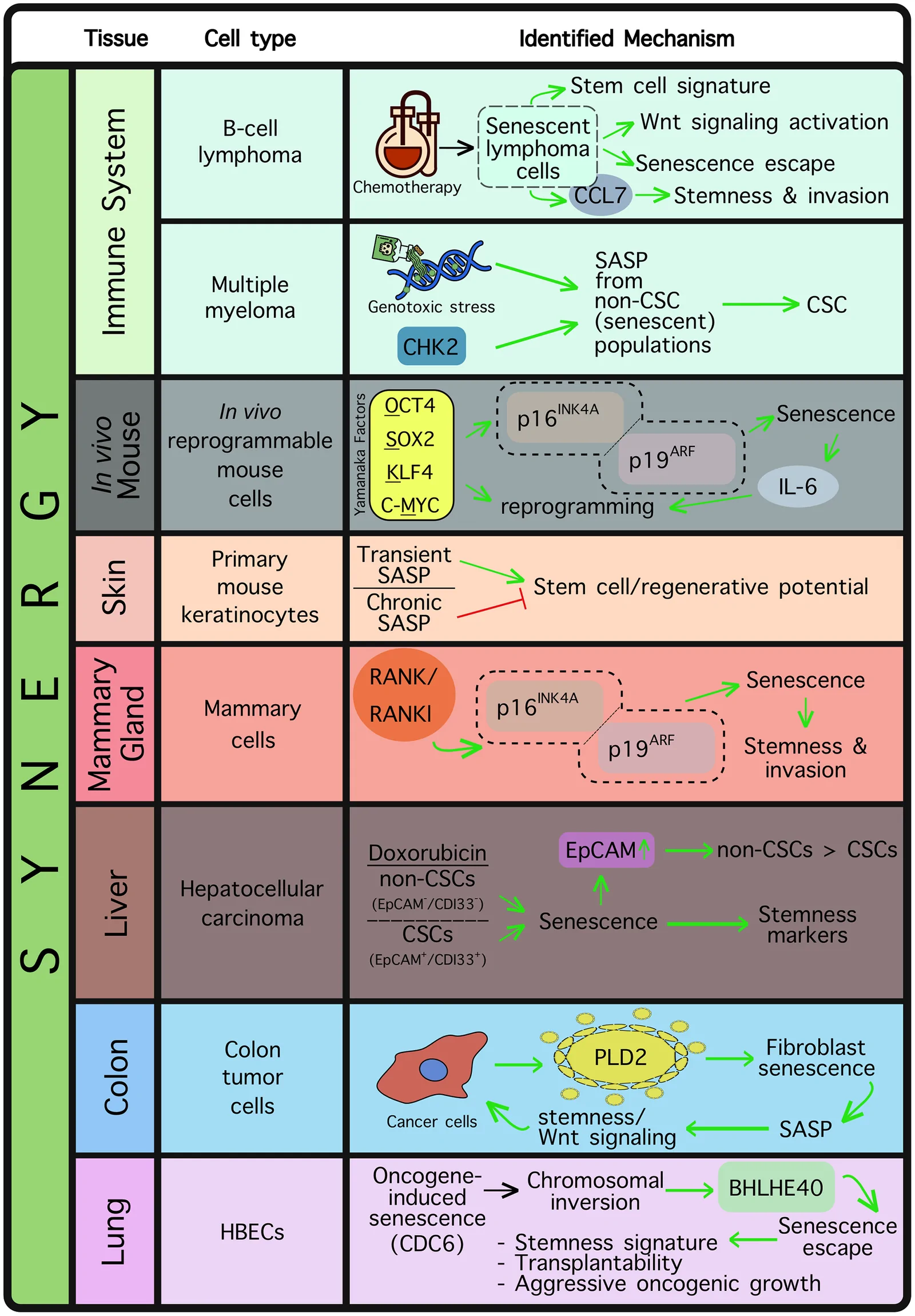
**Tissue and cell types where a synergistic relationship is reportedly established between senescence and stemness.** Synergy is mostly observed in the case of tissue regeneration or, more widely, in cancer. The identified mechanism(s) are depicted in each case.

Resistance to therapy is a major cause of tumor relapse and treatment failure in refractory/relapsed B-cell non-Hodgkin’s lymphoma (referred to as r/r B-NHL), and therapy-induced senescence is considered to be among the most critical drug resistance mechanisms [135]. In a recent study, Wang et al. (2025) identified a distinct population of memory B-cells expressing high levels of C-C chemokine receptor type 7 (CCR7) following therapy-induced senescence, compared to proliferating or spontaneously senescent lymphoma counterparts [136]. CCR7 levels were found strongly augmented in r/r B-NHL patients, an observation linked to poor patient outcome [136]. Mechanistic *in vitro* work showed that CC Motif Chemokine Ligand 21 (CCL21) required CCR7 to drive invasion of B-NHL cells, while CCR7 inhibition limited the invasive properties of these cells after receiving drug treatment [136]. Not surprisingly, B-NHL cells expressing high CCR7 levels following therapy-induced senescence displayed robust stemness properties, such as stem cell marker upregulation *per se*, colony-forming capacity and assumption of highly migratory properties [136].

The SASP has been found to promote stemness in a number of cellular and animal models [137–140]. In multiple myeloma, it was shown that cancer stem cells (CSCs) emerge following genotoxic stress, which also promotes the SASP [137]. In particular, cancer cells undergoing senescence (identified as non-CSC populations), were found to release chemokines leading to the emergence of CSCs from their neighboring cells [137]. However, inhibition of checkpoint protein 2 (CHK2), a major SASP trigger [141], was found to be sufficient in hampering CSC generation [137]. In another study, Mosteiro et al. (2016) provided evidence that *in vivo* induction of the “Yamanaka” OSKM factors in mice may drive both senescence and reprogramming; two processes potentially regulated by genetic loci residing in close proximity and acting in synergy [139]. Indeed, the authors found that OSKM-mediated senescence may require the Ink4a/Arf genetic locus, and upon additional expression of IL-6, a permissive environment for reprogramming is created [139]. In fact, IL-6 has been also shown to promote *in vitro* reprogramming to iPSCs [142]. These findings are in keeping with the general observation that reprogramming is accompanied by replication stress, a driver of senescence [143, 144]. SASP was additionally identified as a major factor contributing to cell plasticity and stemness in primary mouse keratinocytes [140]. Specifically, transient exposure of keratinocytes to SASP led to upregulation of stem cell markers and increase of *in vivo* regenerative potential, however chronic SASP exposure resulted in keratinocyte proliferation arrest, thus attenuating the regeneration stimuli [140]. Overall, the above findings suggest that the SASP may hold a beneficial role in facilitating tissue repair and regeneration, but in the context of tumorigenesis it may exacerbate oncogenic growth by promoting cancer stemness.

Rank and its ligand Rankl constitute master regulators of mammary gland development, functioning as progesterone mediators in the mammary epithelium, both in rodents and humans [145–147]. It is known that Rank induction in the mammary gland under the mouse mammary tumor virus (MMTV) promoter blocks mammary epithelial differentiation and drives stem cell expansion, characterized by an enhanced capacity to reconstitute the mammary gland [148]. Interestingly, Benitez et al. (2021) identified Rank overexpression or Rankl supplementation as factors promoting senescence, via the p16^INK4A^/p19^ARF^ tumor suppressors [149]. Rank-mediated senescence was, however, shown to also drive stemness, and despite an initial tumor growth delay (as a direct consequence of senescence), the tumor subsequently displayed clear metastatic features [149]. As mammary tumors present elevated levels of Rank, these findings are potentially clinically relevant, providing information regarding the therapeutic window where blocking Rank signaling may be beneficial for breast cancer patients. Hence, Rank inhibitors have already started to be exploited in the arena of breast cancer prevention and treatment [149].

Senescence-related stemness was investigated in EpCAM+/CD133+ liver CSC and EpCAM-/CD133- non-CSC populations in the HuH7 immortalized human liver cancer cell line [150]. The authors found that doxorubicin treatment triggered senescence in both CSC and non-CSC populations, however this was accompanied by a marked increase in the expression levels of the SOX2, KLF4 and c-MYC reprogramming factors, as well as of stemness markers [150]. Intriguingly, doxorubicin-mediated senescence conferred a significant increase in the epithelial cell adhesion marker EpCAM in the non-CSC population, suggesting a process of senescence-associated reprogramming into a CSC-like population [150]. Moreover, Wnt/β-catenin pathway target genes were found upregulated in “reprogrammed” cells, whereas blocking of the pathway drastically limited all stem cell features [150]. The authors’ findings highlight the importance of evaluating the potential effects of therapy-induced senescence in hepatocellular carcinomas, in order to avoid the emergence of stemness properties accompanying senescence induction. Additionally, they propose that senescent populations are eliminated timely with the use of potent senolytics [150].

Cancer cells constantly communicate with their surrounding microenvironment, which readily impacts tumor development. Phospholipase D2 (PLD2), known to be involved in proliferation and metastasis [151], was found to be significantly upregulated in colon tumors and released by cancer cells in their microenvironment [152]. Interestingly, cancer-cell released PLD2 was also identified as a senescence-inducing player in neighboring fibroblasts [152]. Senescent fibroblasts subsequently displayed SASP, which led back to an increase of stemness features in cancer cells, including activation of Wnt signaling. This indirect regulation of cancer cell activity indicates the presence of a feedback loop in which cancer cells enhance their own tumorigenicity via inducing paracrine senescence in their microenvironment [152]. The proposed mechanism orchestrated by endogenously increased PLD2 expression in colon cancer cells may provide an additional explanation for the high levels of Wnt pathway activation generally encountered in colon cancer [152].

Senescent cells must be timely removed by immune system components in order to avoid the negative effects accompanying their prolonged presence [1, 26, 153]. In the context of oncogene-induced senescence (OIS), using a human bronchial epithelial cell (HBEC) system carrying an inducible copy of the *CDC6* oncogene, Zampetidis et al. (2021) identified a chromosomal inversion harboring the circadian transcription factor BHLHE40 genetic locus [25]. That inversion was deemed sufficient to activate BHLHE40 and initiate senescence escape, with escaped cells acquiring a significantly more aggressive set of tumorigenic features. Specifically, escaped cells accelerated their proliferation potential and were capable of yielding tumors upon transplantation into nude mice, while their gene expression profiling uncovered a mixed stemness signature consisting of embryonic, epithelial, mesenchymal and MYC-related markers [25]. Importantly, halting CDC6 induction did not reverse the phenotype, implying a permanent establishment of these stemness properties [25]. These observations complement previous findings where Cdc6 induction contributed to EMT by downregulating E-cadherin transcription and activated replication origins [154]. Similarly, prolonged p21^WAF1/Cip1^ expression is sufficient to drive escape from senescence by fueling replication stress, thereby driving genomic instability [22, 155].

## CONCLUSIONS

Given that senescence is intrinsically characterized by proliferation arrest, while stemness refers to an inherent self-renewal capacity and production of differentiated progeny, the two cellular states are often perceived as mutually exclusive. Indeed, in this review we present an accumulation of evidence where the establishment of senescence may impose a barrier to stemness during natural processes such as aging, and upon reversing this functional competition (e.g. via genetic or pharmacological interventions) cell fate may change, as shown in multiple cell types and tissues such as MSCs (PMSCs, BMSCs or ADSCs), muscle satellite cells, dental stem cells or pancreatic β-cells. Interestingly, apart from tissue regeneration, extensive functional synergy between the two states is widely observed in cancer, because the “dark side of senescence” may promote tumorigenic traits through senescent cell paracrine activity or even escape from the senescent state itself. Such activity was found to occur at least in B-cell lymphomas, liver, colon and lung cancer.

It needs to be highlighted that senescence detection in the contexts described above may have occasionally relied on questionable methodologies, especially regarding accuracy. For example, SA-β-Gal assays (including SPiDER-βGal) may suffer from increased background signal or be less suitable for *in vivo* imaging due to limited penetration, autofluorescence or even compromised compound stability [156, 157].

Identifying the clinical settings where targeting senescence may stall tumor growth or stimulate tissue regeneration is of cardinal importance, as it appears that the senescence-stemness interplay is a complex one and vastly dependent on tissue type. Along these lines, promising senolytic-based tools have now been developed in the context of age-related disease and tissue repair [1, 7, 46, 158–160]. However, classical senolytic approaches are still under active clinical evaluation and implementation limitations and concerns have often been raised upon their use in clinical trials [159]. Similarly to those approaches, novel senolytic tools will also have to be carefully evaluated with the use of validated biomarkers and assessed against a clear benefit-risk ratio for patients through appropriate patient stratification, and clear safety/efficacy evidence in adequately powered clinical studies.
